# Effect of fine particulate matter exposure on gestational diabetes mellitus risk: a retrospective cohort study

**DOI:** 10.1093/eurpub/ckae094

**Published:** 2024-05-23

**Authors:** Zhenyan Wan, Shandan Zhang, Guiying Zhuang, Weiqi Liu, Cuiqing Qiu, Huiqin Lai, Weiling Liu

**Affiliations:** Division of Neonatology, The Maternal and Children Health Care Hospital (Huzhong Hospital) of Huadu, Guangzhou, Guangdong, People’s Republic of China; Division of Neonatology, The Maternal and Children Health Care Hospital (Huzhong Hospital) of Huadu, Guangzhou, Guangdong, People’s Republic of China; Division of Neonatology, The Maternal and Children Health Care Hospital (Huzhong Hospital) of Huadu, Guangzhou, Guangdong, People’s Republic of China; Department of Clinical Laboratory, The Maternal and Children Health Care Hospital (Huzhong Hospital) of Huadu, Guangzhou, Guangdong, People’s Republic of China; Medical Information Office, The Maternal and Children Health Care Hospital (Huzhong Hospital) of Huadu, Guangzhou, Guangdong, People’s Republic of China; Department of Clinical Laboratory, Guanzhou Yuexiu Liurong Community Health Service Center, Guangzhou, Guangdong, People’s Republic of China; Department of Clinical Laboratory, Foshan Fosun Chancheng Hospital, Foshan, Guangdong, People’s Republic of China

## Abstract

**Background:**

The literature on the association between fine particulate matter (PM_2.5_) exposure and gestational diabetes mellitus (GDM) risk has focused mainly on exposure during the first and second trimesters, and the research results are inconsistent. Therefore, this study aimed to investigate the associations between PM_2.5_ exposure during preconception, the first trimester and second trimester and GDM risk in pregnant women in Guangzhou.

**Methods:**

A retrospective cohort study of 26 354 pregnant women was conducted, estimating PM_2.5_, particulate matter with a diameter >10 µm (PM_10_), sulphur dioxide (SO_2_), carbon monoxide (CO) and ozone (O_3_) exposure during preconception and the first and second trimesters. Analyses were performed using Cox proportional hazards models and nonlinear distributed lag models.

**Results:**

The study found that exposure to PM_2.5_ or a combination of two pollutants (PM_2.5_+PM_10_, PM_2.5_+SO_2_, PM_2.5_+CO and PM_2.5_+O_3_) was found to be significantly associated with GDM risk (*P* < 0.05). In the second trimester, with significant interactions found for occupation and anaemia between PM_2.5_ and GDM. When the PM_2.5_ concentrations were ≥19.56, ≥25.69 and ≥23.87 μg/m^3^ during preconception and the first and second trimesters, respectively, the hazard ratio for GDM started to increase. The critical window for PM_2.5_ exposure was identified to be from 9 to 11 weeks before conception.

**Conclusions:**

Our study results suggest that PM_2.5_ exposure during preconception and the first and second trimesters increases the risk of GDM, with the preconception period appearing to be the critical window for PM_2.5_ exposure.

## Introduction

Gestational diabetes mellitus (GDM) is a common complication of pregnancy. According to the latest International Diabetes Federation (IDF) report, the global prevalence of GDM is estimated to be 16.7%, affecting more than 21 million live births; the prevalence of GDM in China is 8.6%, affecting more than 1.46 million live births.^1^ Studies have shown that pregnant women with GDM have a 6.43 times greater risk of developing type 2 diabetes than pregnant women without GDM are, and this risk increases over time.^2^ During pregnancy, elevated blood glucose levels can cause excess sugar to cross the placenta from the mother to the foetus, resulting in macrosomia.^3^ In addition, GDM is associated with the risk of heart disease and metabolic disorders in both the baby and the mother later in life.^4^

As global concern about the effects of air pollution on health has increased, there has been growing interest in investigating the association between exposure to fine particulate matter (PM_2.5_) and GDM risk among researchers. Due to the presence of biological and organic components such as metals, polycyclic aromatic hydrocarbons, carbonaceous particles and other organic compounds, PM_2.5_ can cause oxidative stress in the body.^5^ The imbalance between reactive oxygen species production and antioxidant defence in the body is thought to cause changes in the insulin signalling pathway, leading to abnormal glucose metabolism.^6^ An increasing number of studies have shown a strong link between PM_2.5_ exposure and GDM risk.^5^^,^^7^^,^^8^ Long-term exposure to particulate matter is associated with increased blood glucose and lipid levels.^9^ Studies have also shown that both high and low concentrations of PM_2.5_ during the first and second trimesters is associated with an increased risk of GDM.^10^^,^^11^ Although some studies have shown nonsignificant associations between PM_2.5_ exposure and metabolic outcomes, they still suggest an association between high PM_2.5_ concentrations and an increased likelihood of glucose intolerance.^12^

Guangzhou is one of the most economically developed regions in China. It is located in an oceanic subtropical monsoon climate region, and its rapid economic development and urbanization have led to serious air quality challenges. Although a previous study by Liu et al.^13^ found a positive association between air pollution exposure in the first trimester of pregnancy and GDM between 2015 and 2018, it is worth noting that since 2020, the concentration of PM_2.5_ in Guangzhou has reached the secondary standard set by the World Health Organization for four consecutive years (<25 μg/m^3^), which necessitates a re-evaluation of the relationship between PM_2.5_ exposure and GDM risk. In this study, we analyzed the demographic records of 26 354 pregnant women admitted to the Guangzhou from 2018 to 2023. We examined the association between preconception, first- and second-trimester PM_2.5_ exposure and GDM risk. In addition, we investigated the critical window of exposure regarding the association between PM_2.5_ exposure and GDM risk.

## Methods

### Study population

We used a retrospective cohort study design and collected data from pregnant women admitted to the obstetrics department of the Maternal and Children Health Care Hospital of Huadu in Guangzhou, China. The data were obtained from the electronic medical records management system of the hospital. This hospital is a Grade A tertiary level speciality hospital for women and children. The Grade A designation represents the highest level of medical care in the Chinese health care system, and the hospital primarily serves residents of Guangzhou.

The study included a total of 30 078 pregnant women who delivered between 28 November 2018 and 1 February 2023 based on electronic medical records. To minimize the potential impacts on the diagnosis of GDM, we excluded pregnant women who lived outside Guangzhou, those with twin pregnancies, those with diabetes before pregnancy, those with hypertension before pregnancy, and those who underwent *in vitro* fertilization and artificial insemination, among other possible factors. Overall, we included 26 354 pregnant women in the study (Supplementary figure S1). It is worth noting that we anonymized the information of the study participants; therefore, it was not necessary to obtain informed consent. In addition, this study was approved by the Ethics Committee of Maternal and Children Health Care Hospital of Huadu (no. 2024-001), which waived the requirement for informed consent since the study used de-identified information.

### Diagnosis of GDM

The diagnosis of GDM was obtained from electronic medical records, and all participants were diagnosed with GDM based on the 10th revision of the International Classification of Diseases (ICD-10). Each subject underwent an oral glucose tolerance test after at least 8 hours of fasting between the 24th and 28th weeks of pregnancy. During the test, the participant consumed 300 ml of liquid containing 75 g of glucose orally within 5 min. Blood glucose levels were measured before and 1 and 2 h after glucose ingestion. At these time points, a pregnant woman’s blood glucose levels should be below 5.1, 10.0 and 8.5 mmol/l (92, 180, 153 mg/dl), respectively. GDM was diagnosed if any of the blood glucose levels met or exceeded the above criteria.

### Assessment of the PM_2.5_ concentration

We obtained real-time PM_2.5_ concentrations monitored by 10 automatic air quality monitoring stations (Supplementary table S1) at the national level in Guangzhou from the website of Wang Xiaolei (https://quotsoft.net/air/). The daily average PM_2.5_ concentration in Guangzhou was calculated using real-time data from 10 monitoring stations. Wang Xiaolei's website collects nationwide air quality data from the National Environmental Monitoring Centre's National Urban Air Quality Real-time Publishing Platform, which is updated daily.

Based on the calculation of the last menstrual period date according to the infant's birth date and gestational week, we evaluated the average PM_2.5_ exposure concentration during each trimester using the time-varying average concentration method. Based on previous studies,^14^^,^^15^ we calculated the average exposure concentration during three predefined windows: (i) preconception (12 weeks before conception), (ii) the first trimester (1–13 weeks) and (iii) the second trimester (14–28 weeks). To determine the critical exposure windows, we also generated weekly average PM_2.5_ exposure concentrations throughout the follow-up period to assess the effect of PM_2.5_ exposure on GDM risk.

### Covariates

Covariates were identified based on existing studies^16–18^ and information obtained from the electronic medical records management system. We preselected potential confounding factors, including age, occupation, blood type, anaemia, nonprimiparous, eclampsia, hypertension during pregnancy, vaginitis, adverse reproductive history, and daily real-time average concentrations of particulate matter >10 μm (PM_10_), sulphur dioxide (SO_2_), carbon monoxide (CO) and ozone (O_3_). The participants self-reported their occupation type (civil servant, employee, professional, freelancer, self-employed, unemployed, etc.) at the time of enrolment, as well as whether they were primiparous or multiparous (had already delivered more than one child), their blood type (A, B, O, AB), anaemia, eclampsia (mild, moderate, severe), gestational hypertension, vaginal infection, and history of adverse pregnancy outcomes. Occupation was reclassified into three categories: employed, self-employed and other. Data on the daily real-time average concentrations of PM_10_, SO_2_, CO and O_3_ were collected from the same air pollution monitoring stations as those used for PM_2.5_.

### Statistical analysis

The study participants were divided into two groups according to whether they had GDM: the non-GDM group and the GDM group. Categorical variables are presented as case numbers and percentages (%), while continuous variables are presented as medians (*P*_25_, *P*_75_). The chi-square test was used for categorical variables, the Wilcoxon rank-sum test was used for continuous variables, and Spearman correlations were used for PM_2.5_, PM_10_, SO_2_, CO and O_3_ concentrations. We used the Cox proportional hazards model to assess the effect of PM_2.5_ exposure during preconception, the first trimester and the second trimester on GDM risk, adjusting for potential confounders. These confounders included age, occupation, nonprimiparous, blood type, anaemia, eclampsia, hypertension, vaginitis and adverse reproductive history, and we used restricted cubic spline analysis with 4 nodes (i.e. at the 5th, 35th, 65th and 95th centiles) to determine the relationship between PM_2.5_ exposure and GDM risk. To assess the combined effects of exposure to these two pollutants on GDM risk, we included the concentrations of PM_10_, SO_2_, CO and O_3_ in the PM_2.5_ and GDM models. In addition, we performed subgroup analyses of PM_2.5_ exposure and GDM risk to determine the effect of different factors on the association between PM_2.5_ exposure and GDM risk.

To determine the critical window for PM_2.5_ exposure, we evaluated the effect of PM_2.5_ exposure on GDM risk using the distributed lag nonlinear model (DLNM) nested Cox regression method. First, we calculated the average weekly concentration of PM_2.5_ and the average concentrations of PM_10_, SO_2_, CO and O_3_ from 12 weeks preconception to the 28th week of pregnancy for each study participant. The termination time for GDM detection was set at 28 weeks gestation, and the maximum lag time was from 12 weeks preconception to the 28th week of pregnancy (i.e. 0:39). Using the occurrence of GDM as the dependent variable, we used the cross-basis of PM_2.5_ exposure as the independent variable, with a linear function for the exposure–response dimension and a natural spline function for the exposure–lag dimension, with equal spacing for the nodes and 5 degrees of freedom. We also adjusted for the effects of PM_10_, SO_2_, CO and O_3_ concentrations and potential confounders.

All the statistical tests were two-sided, and a *P* < 0.05 was used to indicate statistical significance. Except for the subgroup statistical analysis, which was performed with STATA 16.0 software, all the other statistical analyses were performed with R (version 4.3.2) using the ‘survival’, ‘dlnm’ and ‘rcs’ packages.

## Results

Table 1 presents the baseline characteristics of the study population. Out of the 26 354 individuals included in the study cohort, 4401 were diagnosed with GDM, accounting for 16.7% of the total cohort. The results of the χ^2^ test demonstrated significant differences (*P* < 0.05) between the non-GDM and GDM groups in terms of age, occupation, nonprimiparous, eclampsia, hypertension during pregnancy and adverse reproductive history. Additionally, the nonparametric test results indicated that there were statistically significant differences (*P *<* *0.05) in the levels of air pollutants (PM_2.5_, SO_2_ and O_3_) between the non-GDM and GDM groups.

**Table 1:** Baseline characteristics of the study participants between 2018 and 2023

Variable	Non-GDM (*n *=* *21 953)	GDM (*n *=* *4 401)	*P*-value
Age, *n*(%)			<0.001
≤ 25 years	4441 (20.23%)	472 (10.72%)	
26–30 years	9396 (42.80%)	1546 (35.13%)	
31–35 years	6000 (27.33%)	1555 (35.33%)	
>35 years	2116 (9.64%)	828 (18.81%)	
Race, *n*(%)			0.334
Han	21 468 (97.79%)	4314 (98.02%)	
Other^a^	485 (2.21%)	87 (1.98%)	
Occuption, *n*(%)			<0.001
Employed^b^	12 803 (58.32%)	2433 (55.28%)	
Self-employed^c^	1277 (5.82%)	287 (6.52%)	
Other^d^	7873 (35.86%)	1681 (38.20%)	
Marital status, *n*(%)			0.998
Married	21 175 (96.46%)	4245 (96.46%)	
Non-married	778 (3.54%)	156 (3.54%)	
Blood type, *n*(%)			0.424
Type A	5996 (27.31%)	1152 (26.18%)	
Type B	5515 (25.12%)	1133 (25.74%)	
Type O	8968 (40.85%)	1808 (41.08%)	
Type AB	1474 (6.71%)	308 (7.00%)	
Infant gender, *n*(%)			0.486
Male	11 696 (53.28%)	2370 (53.85%)	
Female	10 257 (46.72%)	2031 (46.15%)	
Anaemia, *n*(%)			0.982
No	13 946 (63.53%)	2795 (63.51%)	
Yes	8007 (36.47%)	1606 (36.49%)	
Nonprimiparous, *n*(%)			<0.001
No	15 152 (69.02%)	2710 (61.58%)	
Yes	6801 (30.98%)	1691 (38.42%)	
Eclampsia, *n*(%)			<0.001
No	21 807 (99.33%)	4339 (98.59%)	
Yes	146 (0.67%)	62 (1.41%)	
Thyroid in pregnancy, *n*(%)			0.058
No	21 007 (95.69%)	4183 (95.05%)	
Yes	946 (4.31%)	218 (4.95%)	
Hypertension during pregnancy, *n*(%)			<0.001
No	21 543 (98.13%)	4244 (96.43%)	
Yes	410 (1.87%)	157 (3.57%)	
Vaginitis, *n*(%)			0.165
No	20 679 (94.20%)	4169 (94.73%)	
Yes	1274 (5.80%)	232 (5.27%)	
Adverse reproductive history, *n*(%)			<0.001
No	19 969 (90.96%)	3825 (86.91%)	
Yes	1984 (9.04%)	576 (13.09%)	
Air pollution, medians (*P*_25_, *P*_75_)			
PM_2.5_, μg/m^3^	27.25 (22.89–29.86)	26.60 (22.69–29.43)	<0.001
PM_10_, μg/m^3^	48.56 (42.85–51.42)	48.10 (42.70–51.77)	0.088
SO_2_, μg/m^3^	7.03 (6.53–7.47)	7.00 (6.47–7.37)	<0.001
CO, mg/m^3^	0.77 (0.74–0.80)	0.77 (0.74–0.80)	0.162
O_3_, μg/m^3^	55.11 (50.68–58.50)	55.32 (51.12–58.91)	<0.001

a Other, Miao, Hui, Tujia, Bai, Zhuang, Yao, etc.

b Employed, professional and technical personnel, civil servants, business managers, etc.

c Self-employed, freelancers, self-employed.

d Other, Farmers, unemployed, students, others.

The Spearman correlation coefficients indicate that PM_2.5_ is positively correlated with PM_10_, SO_2_ and CO, while it has a negative correlation with O_3_. However, there is no obvious correlation between PM_2.5_ and the combination of PM_10_, SO_2_, CO and O_3_ (Supplementary table S2).

Table 2 shows the effects of single-pollutant and double-pollutant PM_2.5_ exposure on GDM risk. After we adjusted for confounding factors in the single-pollutant PM_2.5_ models, we found that there was a statistically significant association between PM_2.5_ exposure and the occurrence of GDM during preconception, the first trimester and the second trimester (*P* < 0.05). The results showed that for each unit increase in the PM_2.5_ exposure concentration from preconception to the second trimester, the risk of GDM increased from 4.2% [95% confidence interval (CI): 1.037–1.046] to 6.7% (95% CI: 1.063–1.072). According to the double-pollutant models, exposure to PM_2.5_+PM_10_, PM_2.5_+SO_2_, PM_2.5_+CO and PM_2.5_+O_3_ was significantly associated with GDM risk (*P *<* *0.05). The results showed that the risk of GDM was the highest in women exposed to PM_2.5_+PM_10_, reaching 61.4% (95% CI: 1.572–1.657) in the second trimester. For PM_2.5_+SO_2_ and PM_2.5_+O_3_ exposure, the risk of GDM gradually increased with increasing weeks of gestation. For PM_2.5_+CO exposure, the highest risk of GDM in the first trimester was 8.1% (95% CI: 1.074–1.089).

**Table 2:** The effects of single-pollutant and double-pollutant PM_2.5_ exposure on GDM risk, 2018–23

Models	Crude		Adjusted^a^	
	*HR* (95%*CI*)	*P*	*HR* (95%*CI*)	*P*
Single-pollution				
PM_2.5_				
Preconception	1.047 (1.043–1.052)	<0.001	1.042 (1.037–1.046)	<0.001
First trimester	1.048 (1.043–1.053)	<0.001	1.046 (1.042–1.051)	<0.001
Second trimester	1.066 (1.061–1.071)	<0.001	1.067 (1.063–1.072)	<0.001
Double-pollution				
PM_2.5_ + PM_10_				
Preconception	1.495 (1.470–1.520)	<0.001	1.476 (1.451–1.501)	<0.001
First trimester	1.499 (1.469–1.529)	<0.001	1.461 (1.432–1.491)	<0.001
Second trimester	1.636 (1.593–1.680)	<0.001	1.614 (1.572–1.657)	<0.001
PM_2.5_ + SO_2_				
Preconception	1.027 (1.019–1.035)	<0.001	1.016 (1.008–1.024)	<0.001
First trimester	1.060 (1.051–1.069)	<0.001	1.046 (1.037–1.055)	<0.001
Second trimester	1.112 (1.101–1.123)	<0.001	1.105 (1.094–1.116)	<0.001
PM_2.5_ + CO				
Preconception	1.070 (1.063–1.077)	<0.001	1.064 (1.057–1.071)	<0.001
First trimester	1.081 (1.073–1.088)	<0.001	1.081 (1.074–1.089)	<0.001
Second trimester	1.054 (1.047–1.062)	<0.001	1.056 (1.049–1.064)	<0.001
PM_2.5_ + O_3_				
Preconception	1.047 (1.043–1.052)	<0.001	1.042 (1.038–1.047)	<0.001
First trimester	1.049 (1.044–1.053)	<0.001	1.047 (1.042–1.052)	<0.001
Second trimester	1.067 (1.063–1.072)	<0.001	1.070 (1.065–1.075)	<0.001

a Adjusted for age, occupation, blood type, anaemia, nonprimiparous, eclampsia, hypertension during pregnancy, vaginitis, adverse reproductive history.

HR, hazard ratio; 95% CI, 95% confidence interval.

In the preconception period, a significant association between PM_2.5_ exposure and GDM risk was found in the occupation and thyroid disease subgroups, with significant interactions observed in the age, occupation, anaemia and nonprimiparous subgroups. In the first trimester, a significant association between PM_2.5_ exposure and GDM risk was observed in the age, hypertension during pregnancy and adverse reproductive history subgroups, with interactions observed in nonprimiparous women. In the second trimester, a significant association was observed in the age, occupation, anaemia, eclampsia and adverse reproductive history subgroups, with significant interactions found for occupation and anaemia (Supplementary table S3).

**Figure 1:**
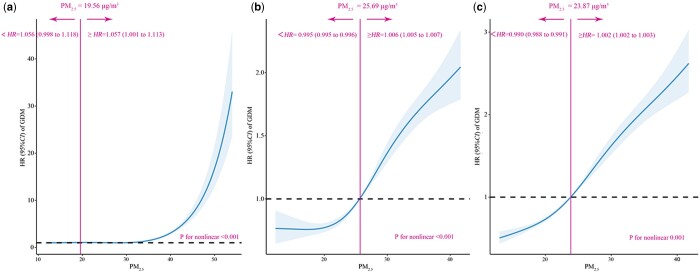
Association of predicted PM_2.5_ exposure with GDM risk, 2018-2023. (a) The preconception period. (b) The first trimester. (c) The second trimester. *HR*, hazard ratio. *95% CI*, 95% confidence interval. Hazard ratios are shown as solid lines, and 95% CIs are shown as shaded areas

A nonlinear relationship between PM_2.5_ exposure and GDM risk was observed, as shown in figure 1. The PM_2.5_ concentrations were ≥19.56, ≥25.69 and ≥23.87 μg/m^3^ in the preconception period and first and second trimesters, respectively; the hazard ratio (HR) for GDM started to increase; and the HR of GDM for each standard deviation increase in the predicted PM_2.5_ concentration were 5.7% (95% CI: 1.001–1.113), 0.6% (95% CI: 1.005–1.007) and 0.2% (95% CI: 1.002–1.003), respectively.

**Figure 2:**
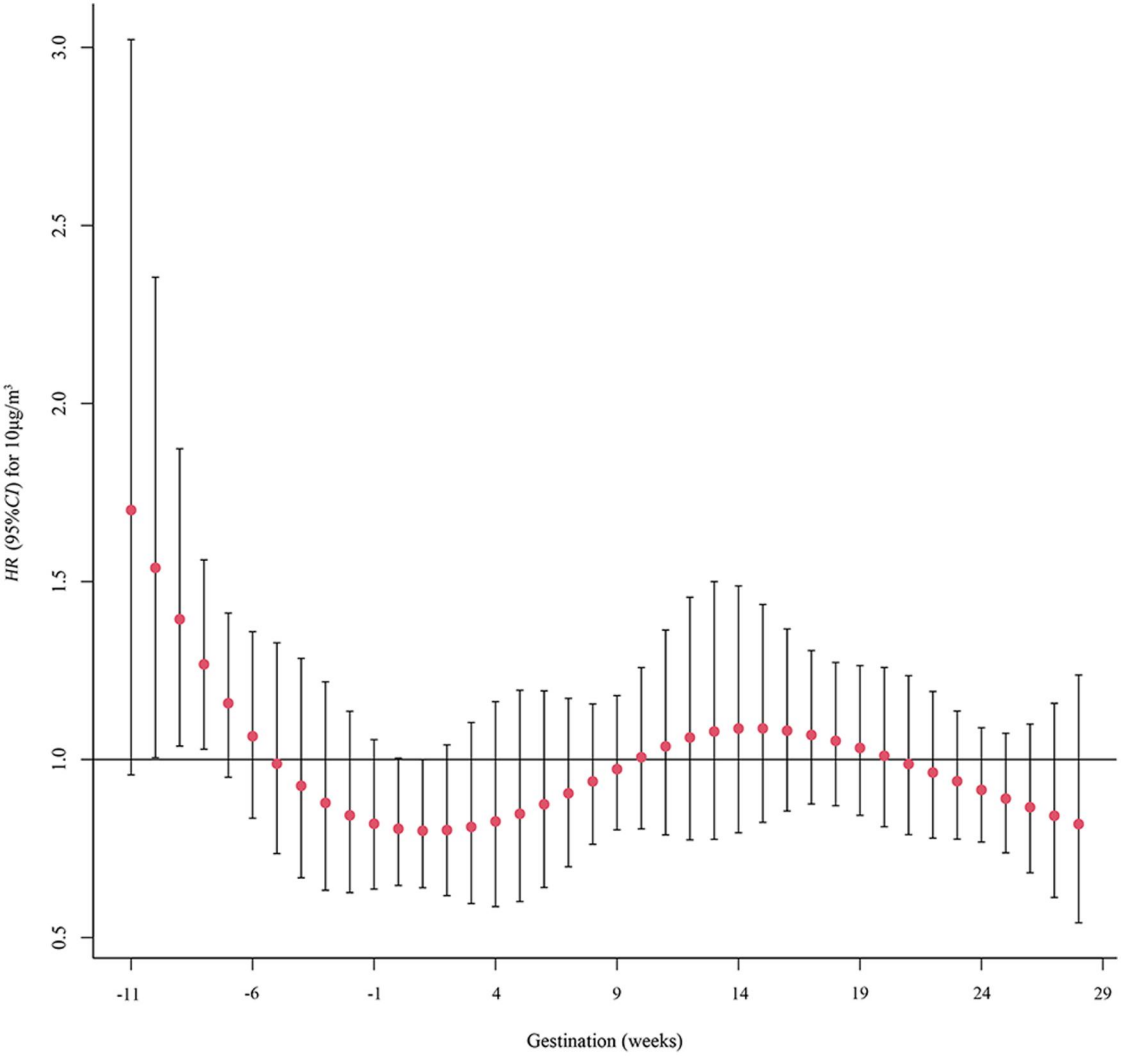
The critical exposure window for the effect of PM_2.5_ exposure on GDM risk, 2018–23. *HR*, hazard ratio. *95% CI*, 95% confidence interval

The risk of GDM associated with PM_2.5_ exposure is depicted in figure 2. The risk of GDM was associated with PM_2.5_ exposure at 9–11 weeks before conception, with the strongest association observed at week 11, with a 56.6% (95% CI: 1.035–2.368) increased risk of GDM for each 10 µg/m^3^ increase.

## Discussion

We conducted a study on the association between PM_2.5_ exposure and GDM risk during different stages of pregnancy using vital statistics from Guangzhou and air pollution data from the Chinese Environmental Monitoring Station. After adjusting for potential confounders, both the single-pollutant model and the two-pollutant model showed a significant association between GDM risk and PM_2.5_ exposure during the preconception period, the first trimester, and the second trimester. We also found that the risk of GDM was highest when PM_2.5_ exposure increased by 10 µg/m^3^ in the 11th week before conception. These findings provide new evidence for the association between PM_2.5_ exposure in pregnant women in Guangzhou and the occurrence of GDM.

Most studies on the association between PM_2.5_ exposure and GDM risk have focused on the first and second trimesters,^19–21^ and there is limited research on the association between preconception exposure to PM_2.5_ and GDM risk. A study conducted by Jo et al. in southern California, USA, from 1999 to 2009 revealed a positive association between preconception exposure to PM_2.5_ or PM_2.5_+PM_10_ and GDM risk.^22^ However, exposure to PM_2.5_+O_3_ did not increase the risk of GDM. In contrast, a study conducted between 2002 and 2008 involving 208 695 pregnant women from 12 clinical centres in the USA revealed that preconception exposure to PM_2.5_ did not increase the risk of GDM (HR, 0.97; 95% CI: 0.93–1.02),^23^ which is not entirely consistent with the results of our study. Our study showed a significant association between preconception exposure and an increased risk of GDM in both single- and double-pollutant models. This discrepancy may be due to differences in geographic and environmental factors, as well as differences in race, lifestyle and study period in the reference population. Furthermore, we also found a potentially critical window for the effect of PM_2.5_ exposure on GDM risk from the 9th to the 11th week before conception, which is inconsistent with the findings of previous reports. Zheng et al. conducted a study on the association of exposure to PM_2.5_ and its components with GDM risk and reported that the critical window for the effect of ${\text{SO}}_{4}^{2-}$ in PM_2.5_ on GDM risk ranged from 13 to 24 weeks of gestation.^15^ This may be related to differences in the primary indicators of exposure. Our study used PM_2.5_, whereas previous studies used the ${\text{SO}}_{4}^{2-}$ component of PM_2.5_. Different exposure indicators may have different effects on the critical exposure window for GDM.

Our research findings revealed an association between exposure to PM_2.5_ during the first and second trimesters and the risk of GDM. This finding is consistent with the study conducted by Liang et al. which also revealed an increased risk of GDM with PM_2.5_ exposure.^24^ However, in a study conducted in Massachusetts from 2003–8, Fleisch et al. did not find an association between PM_2.5_ exposure during the first or second trimester and GDM risk. They found an increased risk of GDM with PM_2.5_ exposure only during the second trimester in women under the age of 20 years (OR, 1.36; 95% CI: 1.08–1.70).^25^ The potential reasons for this inconsistency are that the different studies used different adjustment models and included populations with different characteristics. Additionally, differences in survey times and PM_2.5_ exposure assessment methods may also contribute to the consistency. Fleisch et al.'s study covered the period from 2003–8 and used a satellite-based spatiotemporal model for PM_2.5_ exposure assessment, while our study covered the period from 2018 to –23 and used a time-varying concentration approach for PM_2.5_ exposure assessment.

However, the relationship between PM_2.5_ exposure and GDM risk has not been fully investigated. PM_2.5_ is one of the main components of air pollution and has the potential to cause adverse health effects. Research by Janssen et al. in the ENVIRONAGE birth cohort study revealed a significant association between PM_2.5_ exposure during the first trimester and placental DNA methylation (−2.13% per 5 μg/m^3^ increase, 95% CI: −3.71, −0.54%, *P* = 0.009).^26^ A study conducted in Tehran, Iran, revealed a significant correlation between PM_2.5_ exposure in the first trimester and placental methylation,^27^ and placental DNA methylation has been shown to be associated with GDM.^28^^,^^29^ In addition, animal studies have shown that exposure to PM_2.5_ can exacerbate oxidative stress, insulin resistance, inflammation and obesity.^30^^,^^31^ During the prepregnancy period, women often experience weight gain due to an excessive focus on nutrient intake. In normal pregnancy, insulin resistance occurs to meet the nutritional needs of the placenta and foetus, which is usually compensated for by increased insulin secretion and an adaptive increase in pancreatic beta-cell mass.^32^ However, exposure to PM_2.5_ during the preconception period or pregnancy may exacerbate the development of obesity and accelerate the development of physiological insulin resistance and beta-cell dysfunction, thereby accelerating the progression of GDM.^33^^,^^34^ Therefore, from a physiological perspective, maternal exposure to PM_2.5_ during preconception and pregnancy may increase the risk of GDM.

This study has a number of limitations. First, there was a lack of information on activity patterns during pregnancy or home relocation, which may have affected the accurate assessment of the pregnant women’s indoor PM_2.5_ exposure. The lack of this information may reduce the accuracy of the exposure estimates. Second, there may have been some errors in the classification of outcomes based on the diagnosis of GDM in electronic medical records. Studies have shown that the specificity of identifying GDM using discharge data is 98%, with a sensitivity of 71–81%. This may have led to inappropriate grouping of individuals with GDM. In addition, due to the limited information provided by electronic medical records, we could not account for all factors that may be associated with PM_2.5_ exposure and GDM risk, such as the participants' daily activities, prepregnancy and pregnancy body mass index, education level and income level. The absence of these factors may limit a full understanding of the relationship between exposure to air pollution and GDM risk. Previous research has shown that people with lower education and income levels are more likely to live within one mile of pollution sources.^35^ Therefore, there may be some degree of selection bias in the selection of the study participants. Finally, this study assessed PM_2.5_ exposure based on temporal concentration variations but did not assess whether the study subjects had indoor PM_2.5_ exposure. Several studies have shown that although most urban residents use clean energy, a quarter of the population still relies on solid fuels for heating.^36^ This may have led to an underestimation of the association between PM_2.5_ exposure and GDM risk in this study.

In conclusion, our study results confirmed that exposure to PM_2.5_ during preconception, the first trimester and the second trimester increases the risk of GDM in pregnant women, with the preconception period appearing to constitute a window of vulnerability. Our findings may help policy-makers develop appropriate preventive measures to reduce the adverse effects of PM_2.5_ exposure in pregnant women.

## Supplementary Material

ckae094_Supplementary_Data

## Data Availability

The data underlying this article will be shared on reasonable request to the corresponding author.

## References

[1] International Diabetes Federation. *IDF Diabetes Atlas*, 10th edn. Available at: https://diabetesatlas.org/data/en/world/.

[2] Juan J , Sun Y, Wei Y, et al Progression to type 2 diabetes mellitus after gestational diabetes mellitus diagnosed by IADPSG criteria: systematic review and meta-analysis. Front Endocrinol (Lausanne) 2022;13:1012244. 36277725 10.3389/fendo.2022.1012244 PMC9582268

[3] Zheng W , Huang W, Liu C, et al Weight gain after diagnosis of gestational diabetes mellitus and its association with adverse pregnancy outcomes: a cohort study. BMC Pregnancy Childbirth 2021;21:216. 33731035 10.1186/s12884-021-03690-z PMC7971950

[4] Kramer CK , Campbell S, Retnakaran R. Gestational diabetes and the risk of cardiovascular disease in women: a systematic review and meta-analysis. Diabetologia 2019;62:905–14. 30843102 10.1007/s00125-019-4840-2

[5] Wang Q , Liu S. The effects and pathogenesis of PM_2.5_ and its components on chronic obstructive pulmonary disease. Int J Chron Obstruct Pulmon Dis 2023;18:493–506. 37056681 10.2147/COPD.S402122 PMC10086390

[6] Lamb RE , Goldstein BJ. Modulating an oxidative-inflammatory cascade: potential new treatment strategy for improving glucose metabolism, insulin resistance, and vascular function. Int J Clin Pract 2008;62:1087–95. 18489578 10.1111/j.1742-1241.2008.01789.x PMC2440526

[7] Elshahidi MH. Outdoor air pollution and gestational diabetes mellitus: a systematic review and meta-analysis. Iran J Public Health 2019;48:9–19. 30847307 PMC6401584

[8] He D , Wu S, Zhao H, et al Association between particulate matter 2.5 and diabetes mellitus: a meta-analysis of cohort studies. J Diabetes Investig 2017;8:687–96. 10.1111/jdi.12631 PMC5583950 28122165

[9] Chuang KJ , Yan YH, Chiu SY, Cheng TJ. Long-term air pollution exposure and risk factors for cardiovascular diseases among the elderly in Taiwan. Occup Environ Med 2011;68:64–8. 20833756 10.1136/oem.2009.052704

[10] Shen HN , Hua SY, Chiu CT, Li CY. Maternal exposure to air pollutants and risk of gestational diabetes mellitus in Taiwan. Int J Environ Res Public Health 2017;14:1604. 29261145 10.3390/ijerph14121604 PMC5751021

[11] Hu H , Ha S, Henderson BH, et al Association of atmospheric particulate matter and ozone with gestational diabetes mellitus. Environ Health Perspect 2015;123:853–9. 25794412 10.1289/ehp.1408456 PMC4559952

[12] Rammah A , Whitworth KW, Amos CI, et al Air pollution, residential greenness and metabolic dysfunction during early pregnancy in the INfancia y Medio Ambiente (INMA) Cohort. Int J Environ Res Public Health 2021;18:9354. 34501944 10.3390/ijerph18179354 PMC8430971

[13] Liu W-Y , Lu J-H, He J-R, et al Combined effects of air pollutants on gestational diabetes mellitus: a prospective cohort study. Environ Res 2022;204:112393. 34798119 10.1016/j.envres.2021.112393

[14] Zhang H , Zhao Y. Ambient air pollution exposure during pregnancy and gestational diabetes mellitus in Shenyang, China: a prospective cohort study. Environ Sci Pollut Res Int 2021;28:7806–14. 33037545 10.1007/s11356-020-11143-x

[15] Zheng Y , Bian J, Hart J, et al PM_2.5_ constituents and onset of gestational diabetes mellitus: identifying susceptible exposure windows. Atmos Environ 2022;291:119409. 10.1016/j.atmosenv.2022.119409 PMC10162772 37151750

[16] Dastoorpoor M , Khanjani N, Moradgholi A, et al Prenatal exposure to ambient air pollution and adverse pregnancy outcomes in Ahvaz, Iran: a generalized additive model. Int Arch Occup Environ Health 2021;94:309–24. 32936369 10.1007/s00420-020-01577-8

[17] Gong Z , Yue H, Li Z, et al Association between maternal exposure to air pollution and gestational diabetes mellitus in Taiyuan, North China. Sci Total Environ 2023;875:162515. 36868286 10.1016/j.scitotenv.2023.162515

[18] He J , Wang Y, Liu Y, et al Experiences of pregnant women with gestational diabetes mellitus: a systematic review of qualitative evidence protocol. BMJ Open 2020;10:e034126. 10.1136/bmjopen-2019-034126 PMC7044968 32075837

[19] Cheng X , Ji X, Yang D, et al Associations of PM(2.5) exposure with blood glucose impairment in early pregnancy and gestational diabetes mellitus. Ecotoxicol Environ Saf 2022;232:113278. 35131583 10.1016/j.ecoenv.2022.113278

[20] Kang J , Liao J, Xu S, et al Associations of exposure to fine particulate matter during pregnancy with maternal blood glucose levels and gestational diabetes mellitus: potential effect modification by ABO blood group. Ecotoxicol Environ Saf 2020;198:110673. 32361495 10.1016/j.ecoenv.2020.110673

[21] Liu W , Zhang Q, Liu W, Qiu C. Association between air pollution exposure and gestational diabetes mellitus in pregnant women: a retrospective cohort study. Environ Sci Pollut Res Int 2023;30:2891–903. 35941503 10.1007/s11356-022-22379-0

[22] Jo H , Eckel SP, Chen J-C, et al Associations of gestational diabetes mellitus with residential air pollution exposure in a large Southern California pregnancy cohort. Environ Int 2019;130:104933. 31234004 10.1016/j.envint.2019.104933 PMC6684238

[23] Robledo CA , Mendola P, Yeung E, et al Preconception and early pregnancy air pollution exposures and risk of gestational diabetes mellitus. Environ Res 2015;137:316–22. 25601734 10.1016/j.envres.2014.12.020 PMC6204222

[24] Liang W , Zhu H, Xu J, et al Ambient air pollution and gestational diabetes mellitus: an updated systematic review and meta-analysis. Ecotoxicol Environ Saf 2023;255:114802. 36934545 10.1016/j.ecoenv.2023.114802

[25] Fleisch AF , Kloog I, Luttmann-Gibson H, et al Air pollution exposure and gestational diabetes mellitus among pregnant women in Massachusetts: a cohort study. Environ Health 2016;15:40. 26911579 10.1186/s12940-016-0121-4 PMC4765142

[26] Janssen BG , Godderis L, Pieters N, et al Placental DNA hypomethylation in association with particulate air pollution in early life. Part Fibre Toxicol 2013;10:22. 23742113 10.1186/1743-8977-10-22 PMC3686623

[27] Maghbooli Z , Hossein-Nezhad A, Adabi E, et al Air pollution during pregnancy and placental adaptation in the levels of global DNA methylation. PLoS One 2018;13:e0199772. 29979694 10.1371/journal.pone.0199772 PMC6034814

[28] Lizarraga D , Garcia-Gasca T, Lund G, et al Global DNA methylation and miR-126-3p expression in Mexican women with gestational diabetes mellitus: a pilot study. Mol Biol Rep 2023;51:5. 38085382 10.1007/s11033-023-09005-z

[29] Reichetzeder C , Dwi Putra SE, Pfab T, et al Increased global placental DNA methylation levels are associated with gestational diabetes. Clin Epigenetics 2016;8:82. 27462376 10.1186/s13148-016-0247-9 PMC4960714

[30] Sun Q , Yue P, Deiuliis JA, et al Ambient air pollution exaggerates adipose inflammation and insulin resistance in a mouse model of diet-induced obesity. Circulation 2009;119:538–46. 19153269 10.1161/CIRCULATIONAHA.108.799015 PMC3845676

[31] Haberzettl P , O'Toole TE, Bhatnagar A, Conklin DJ. Exposure to fine particulate air pollution causes vascular insulin resistance by inducing pulmonary oxidative stress. Environ Health Perspect 2016;124:1830–9. 27128347 10.1289/EHP212 PMC5132639

[32] Hill DJ. Placental control of metabolic adaptations in the mother for an optimal pregnancy outcome. What goes wrong in gestational diabetes? Placenta. Placenta 2018;69:162–8. 29352600 10.1016/j.placenta.2018.01.002

[33] Kubo A , Ferrara A, Windham GC, et al Maternal hyperglycemia during pregnancy predicts adiposity of the offspring. Diabetes Care 2014;37:2996–3002. 25150158 10.2337/dc14-1438 PMC4207207

[34] Zhao L , Fang J, Tang S, et al PM_2.5_ and serum metabolome and insulin resistance, potential mediation by the gut microbiome: a population-based panel study of older adults in China. Environ Health Perspect 2022;130:27007. 35157499 10.1289/EHP9688 PMC8843086

[35] Mohai P , Lantz PM, Morenoff J, et al Racial and socioeconomic disparities in residential proximity to polluting industrial facilities: evidence from the Americans' Changing Lives Study. Am J Public Health 2009;99(Suppl 3):S649–56. 19890171 10.2105/AJPH.2007.131383 PMC2774179

[36] Chan KH , Lam KBH, Kurmi OP, China Kadoorie Biobank collaborative group, et al Trans-generational changes and rural-urban inequality in household fuel use and cookstove ventilation in China: a multi-region study of 0.5 million adults. Int J Hyg Environ Health 2017;220:1370–81. 28986011 10.1016/j.ijheh.2017.09.010 PMC6675611

