# Supplementary material for: Effect of fine particulate matter exposure on gestational diabetes mellitus risk: a retrospective cohort study
Source: Eur J Public Health. 2024 May 23;34(4):787–93. doi: 10.1093/eurpub/ckae094 (PMC11293809; doi:10.1093/eurpub/ckae094)
Supplement: ckae094_Supplementary_Data [file ckae094_supplementary_data.docx]

**Supplementary Figure and Tables**

**Figure S1** Study selection flowchart for pregnant women, 2018-2023


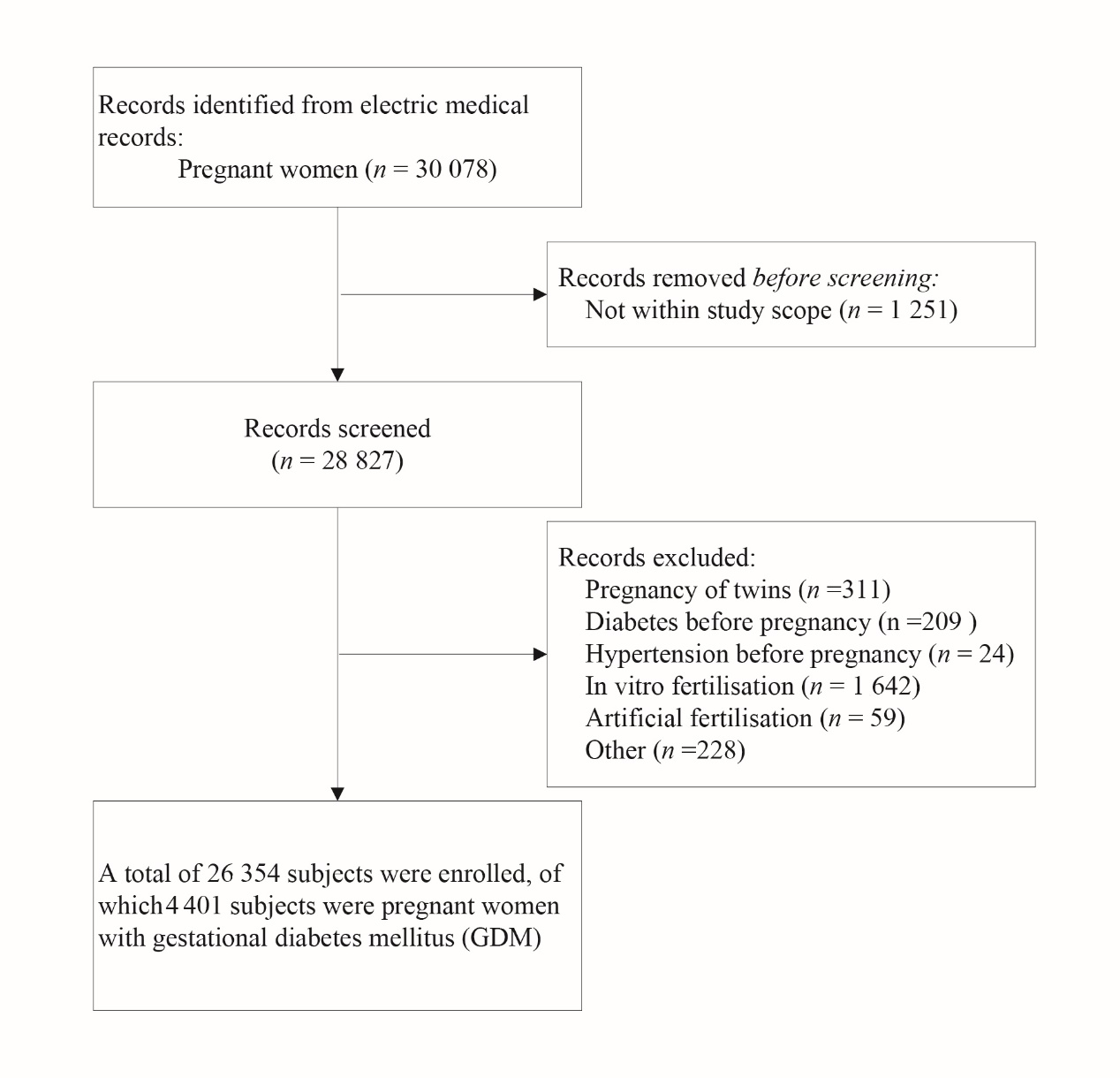


**Table S1** Air quality state-controlled automatic monitoring stations for this study

| Stations ID | Stations Name | Longitude | Latitude |
| --- | --- | --- | --- |
| 1345A | Guangya Zhongxue | 113.2347 | 23.1423 |
| 1346A | Shi Wuzhong | 113.2612 | 23.105 |
| 1348A | Guangdong Shangyuan Daxue | 113.3478 | 23.0916 |
| 1349A | Shi Baiwuzhong | 113.4332 | 23.1047 |
| 1350A | Panyu Zhongxue | 113.3505 | 22.9483 |
| 1351A | Huadong Shifei | 113.2146 | 23.3916 |
| 1352A | Shi Jiancezhan | 113.2597 | 23.1331 |
| 1353A | Jiulong Zhen Zhenlong | 113.5618 | 23.312 |
| 1354A | Luhu | 113.2765 | 23.1544 |

**Table S2** Spearman correlation analysis of PM_2.5_, PM_10_, SO_2_, CO, and O_3_ concentrations during the study period, 2018-2023

|  | PM_2.5_ | PM_10_ | SO_2_ | CO | O_3_ |
| --- | --- | --- | --- | --- | --- |
| PM_2.5_ | 1.000 | 0.840 | 0.661 | 0.686 | -0.049 |
| PM_10_ | 0.840 | 1.000 | 0.762 | 0.521 | 0.386 |
| SO_2_ | 0.661 | 0.762 | 1.000 | 0.048 | 0.396 |
| CO | 0.686 | 0.521 | 0.048 | 1.000 | -0.361^*^ |
| O_3_ | -0.049 | 0.386 | 0.396 | -0.361^*^ | 1.000 |

^*^P <0.05

**Table S3** Subgroup analyses of the association between PM_2.5_ exposure at different times and GDM risk, 2018-2023.

|  | *n* | Preconception | | |  | First trimester | | |  | Second trimester | | |
| --- | --- | --- | --- | --- | --- | --- | --- | --- | --- | --- | --- | --- |
|  |  | *HR* (95%*CI*) | *p*-value | *p-*interaction |  | *HR* (95%*CI*) | *p*-value | *p-*interaction |  | *HR* (95%*CI*) | *p*-value | *p-*interaction |
| Age |  |  |  | 0.020 |  |  |  | 0.901 |  |  |  | 0.867 |
| ≤ 25 years | 4 913 | 1 (Ref) |  |  |  | 1 (Ref) |  |  |  | 1 (Ref) |  |  |
| 26-30 years | 10 942 | 0.826 (0.561-1.217) | 0.335 |  |  | 1.313 (0.894-1.930) | 0.165 |  |  | 1.433 (1.004-2.044) | 0.047 |  |
| 31-35 years | 7 555 | 1.060 (0.718-1.565) | 0.768 |  |  | 1.942 (1.320-2.856) | 0.001 |  |  | 2.013 (1.414-2.867) | <0.001 |  |
| >35 years | 2 944 | 1.334 (0.866-2.056) | 0.192 |  |  | 3.028 (1.985-4.621) | <0.001 |  |  | 2.683 (1.816-3.963) | <0.001 |  |
| Race |  |  |  | 0.144 |  |  |  | 0.452 |  |  |  | 0.468 |
| Han | 25 782 | 1 (Ref) |  |  |  | 1 (Ref) |  |  |  | 1 (Ref) |  |  |
| Other | 572 | 1.600 (0.691-3.704) | 0.273 |  |  | 1.164 (0.514-2.637) | 0.715 |  |  | 0.675 (0.335-1.359) | 0.271 |  |
| Occuption |  |  |  | 0.043 |  |  |  | 0.938 |  |  |  | 0.010 |
| Emplyoee | 15 236 | 1 (Ref) |  |  |  | 1 (Ref) |  |  |  | 1 (Ref) |  |  |
| Freelance | 1 564 | 2.287 (1.434-3.650) | 0.001 |  |  | 0.999 (0.983-1.016) | 0.943 |  |  | 1.199 (0.791-1.817) | 0.392 |  |
| Other | 9 554 | 1.487 (1.166-1.897) | 0.001 |  |  | 0.997 (0.989-1.006) | 0.544 |  |  | 2.011 (1.625-2.488) | <0.001 |  |
| Marital status |  |  |  | 0.517 |  |  |  | 0.862 |  |  |  | 0.789 |
| Married | 25 420 | 1 (Ref) |  |  |  | 1 (Ref) |  |  |  | 1 (Ref) |  |  |
| Non-married | 934 | 0.894 (0.476-1.681) | 0.729 |  |  | 1.160 (0.643-2.092) | 0.623 |  |  | 1.044 (0.598-1.821) | 0.880 |  |
| Blood type |  |  |  | 0.508 |  |  |  | 0.665 |  |  |  | 0.158 |
| Type A | 7 148 | 1 (Ref) |  |  |  | 1 (Ref) |  |  |  | 1 (Ref) |  |  |
| Type B | 6 648 | 1.103 (0.800-1.519) | 0.550 |  |  | 1.056 (0.777-1.435) | 0.727 |  |  | 1.261 (0.954-1.669) | 0.104 |  |
| Type O | 10 776 | 1.006 (0.753-1.344) | 0.968 |  |  | 0.931 (0.706-1.227) | 0.612 |  |  | 1.201 (0.933-1.545) | 0.154 |  |
| Type AB | 1 782 | 0.884 (0.532-1.468) | 0.633 |  |  | 0.971 (0.608-1.55) | 0.901 |  |  | 0.877 (0.573-1.344) | 0.548 |  |
| Infant gender |  |  |  | 0.566 |  |  |  | 0.805 |  |  |  | 0.397 |
| Male | 14 066 | 1 (Ref) |  |  |  | 1 (Ref) |  |  |  | 1 (Ref) |  |  |
| Female | 12 288 | 0.909 (0.721-1.147) | 0.422 |  |  | 1.005 (0.806-1.255) | 0.962 |  |  | 1.063 (0.869-1.301) | 0.550 |  |
| Anemia |  |  |  | 0.008 |  |  |  | 0.487 |  |  |  | <0.001 |
| No | 16 741 | 1 (Ref) |  |  |  | 1 (Ref) |  |  |  | 1 (Ref) |  |  |
| Yes | 9 613 | 1.268 (0.999-1.610) | 0.051 |  |  | 0.985 (0.783-1.239) | 0.896 |  |  | 0.642 (0.521-0.793) | <0.001 |  |
| Non-primary |  |  |  | 0.025 |  |  |  | 0.002 |  |  |  | 0.409 |
| No | 17 862 | 1 (Ref) |  |  |  | 1 (Ref) |  |  |  | 1 (Ref) |  |  |
| Yes | 8 492 | 1.038 (0.817-1.318) | 0.759 |  |  | 1.118 (0.89-1.403) | 0.337 |  |  | 0.833 (0.676-1.027) | 0.088 |  |
| Eclampsia |  |  |  | 0.506 |  |  |  | 0.500 |  |  |  | 0.109 |
| No | 26 146 | 1 (Ref) |  |  |  | 1 (Ref) |  |  |  | 1 (Ref) |  |  |
| Yes | 208 | 1.217 (0.466-3.175) | 0.689 |  |  | 1.292 (0.54-3.092) | 0.564 |  |  | 3.363 (1.389-8.14) | 0.007 |  |
| Thyroid disease in pregnancy |  |  |  | 0.080 |  |  |  | 0.420 |  |  |  | 0.805 |
| No | 25 190 | 1 (Ref) |  |  |  | 1 (Ref) |  |  |  | 1 (Ref) |  |  |
| Yes | 1 164 | 1.710 (1.012-2.890) | 0.045 |  |  | 1.333 (0.797-2.228) | 0.273 |  |  | 1.027 (0.652-1.619) | 0.909 |  |
| Hypertension in pregnancy |  |  |  | 0.910 |  |  |  | 0.779 |  |  |  | 0.657 |
| No | 25 787 | 1 (Ref) |  |  |  | 1 (Ref) |  |  |  | 1 (Ref) |  |  |
| Yes | 567 | 1.730 (0.938-3.191) | 0.079 |  |  | 1.84 (1.043-3.244) | 0.035 |  |  | 1.520 (0.861-2.683) | 0.149 |  |
| Vaginitis |  |  |  | 0.067 |  |  |  | 0.584 |  |  |  | 0.415 |
| No | 24 848 | 1 (Ref) |  |  |  | 1 (Ref) |  |  |  | 1 (Ref) |  |  |
| Yes | 1 506 | 1.236 (0.754-2.207) | 0.401 |  |  | 0.913 (0.552-1.507) | 0.721 |  |  | 0.659 (0.416-1.043) | 0.075 |  |
| Adverse reproductive history |  |  |  | 0.788 |  |  |  | 0.395 |  |  |  | 0.201 |
| No | 23 794 | 1 (Ref) |  |  |  | 1 (Ref) |  |  |  | 1 (Ref) |  |  |
| Yes | 2 560 | 1.283 (0.915-1.799) | 0.148 |  |  | 1.538 (1.115-2.121) | 0.009 |  |  | 1.629 (1.200-2.212) | 0.002 |  |

*HR*, *hazard ratio. 95% CI*, 95% confidence interval.
